# A Large Intraoral Sublingual Schwannoma in a Pediatric Patient: A Case Report

**DOI:** 10.22038/ijorl.2021.44977.2478

**Published:** 2021-09

**Authors:** Santosh Kumar Swain, Smrutipragnya Samal, Somadatta Das, Rabindranath Padhy

**Affiliations:** 1 *Department of Otorhinolaryngology, IMS and SUM Hospital, Siksha “O” Anusandhan University (Deemed to be), K8, Kalinga Nagar, Bhubaneswar-751003, Odisha, India.*; 2 *Central Research Laboratory, IMS and SUM Hospital, Siksha “O” Anusandhan University (Deemed to be), K8, Kalinga N* *.*

**Keywords:** Tongue, Schwannoma, MRI, Oral cavity

## Abstract

**Introduction:**

Schwannoma is a benign neoplasm that arises from Schwannoma cells found in the peripheral nerve sheath. It's a frequent neoplasm in the head and neck area, but it's exceedingly unusual to find it in the mouth. It's a rare occurrence in the oral cavity of the pediatric age group.

**Case Report:**

We present a 12-year-old kid who has had a smooth, firm, and non-tender mass in the sublingual region for the past year. The mass was removed completely using a transoral technique. The diagnosis of sublingual schwannoma was confirmed by histopathological and immunohistochemical testing.

**Conclusion:**

Schwannomas are typically benign and have a good prognosis with a low risk of malignant change. It should be used as a differential diagnostic for sublingual diseases such as ranula and salivary gland lesions. In the case of lingual schwannoma, surgical removal of the tumor is the preferred therapy. The transoral method is the most popular treatment option for sublingual schwannoma.

## Introduction

Schwannoma is a benign neurogenic tumor that originates in the Schwann cells of the peripheral nerve sheath. This is a single, encapsulated tumor that grows slowly. They are exceedingly uncommon, especially in the oral cavity's sublingual region. It is a common tumor in the head and neck area, accounting for 25% to 45% of all schwannomas. However, its presence in the oral cavity (about 1% of the time) is exceedingly rare (1). Any peripheral, cranial (excluding olfactory and ocular), or autonomic neuron containing Schwann cells, the cells that create the myelin coating around the nerve fibers, can cause it. This tumor can develop alone or in combination with neurofibromatosis type 1 (NF1) or type 2 (NF2), as well as schwannomatosis, a genetically inherited illness. The NF2 gene controls Schwann cells and functions as a tumor suppressor (2). In the case of the tongue, the identification of the nerve which gives rise to the nerve is often difficult (hypoglossal, lingual, or glossopharyngeal) due to their proximity (3). Transoral excision of the tumor is the usual therapeutic method. This tumor's recurrence is extremely unusual, as is its malignant change (4). Schwannoma was first described by Verocay in 1910, who coined the term "neuriloma" to describe this benign neurogenic tumor (5). Schwannoma can be found at any age, although most commonly found between the 3rd and 6th decades, and it has no gender preference (4). An instance of a massive intraoral sublingual schwannoma in a juvenile child is presented here.

## Case Report

**Fig 1:**
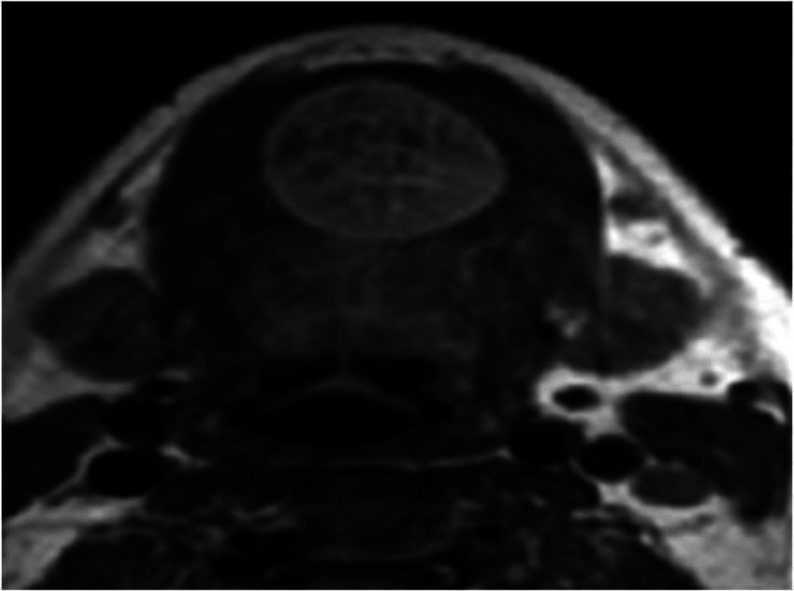
MRI of the tongue revealed the presence of a well-circumscribed nodule on the right anterior tongue, homogeneously and homo-geneously hyperintense on T2WI image

A 12-year-old male child has had a slow-growing, painless mass in the sublingual region for one year. Except for a swelling in the sublingual region, the patient reported no additional symptoms. There was no information about his personal or family medical history. A solid, non-tender lump measuring 4.5x7 cm was discovered in the sublingual region of the tongue during an oral examination. Other clinical tests were within normal ranges, and he showed no indication of neck node growth. An MRI revealed a well-confined ovulated nodular mass in the sublingual region of the tongue that was homogeneously isointense to the muscle on T1W1 and homogeneously hyperintense on T2W1, indicating homogeneous hypervascular enhancement (Fig.1). 

**Fig 2:**
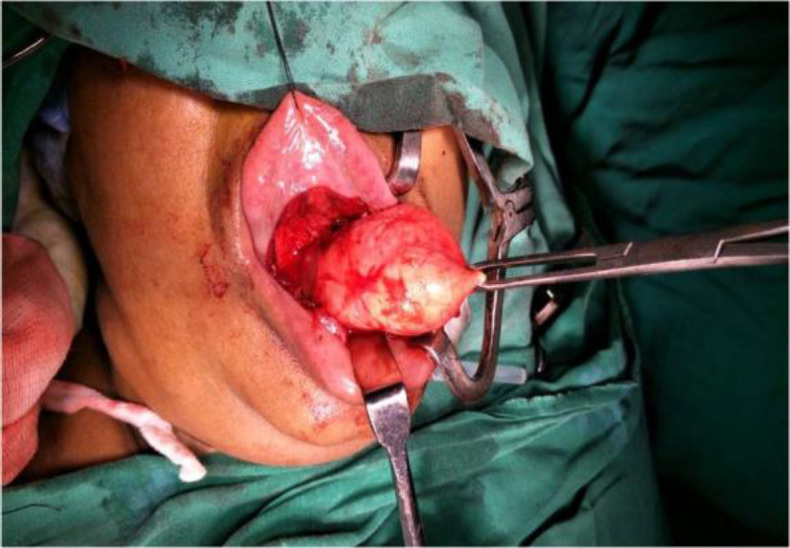
Intra-operative picture of the sublingual schwannoma

This tumor was removed under general anesthesia and sent for histological investigation for confirmation of the diagnosis (Fig.2).

**Fig 3:**
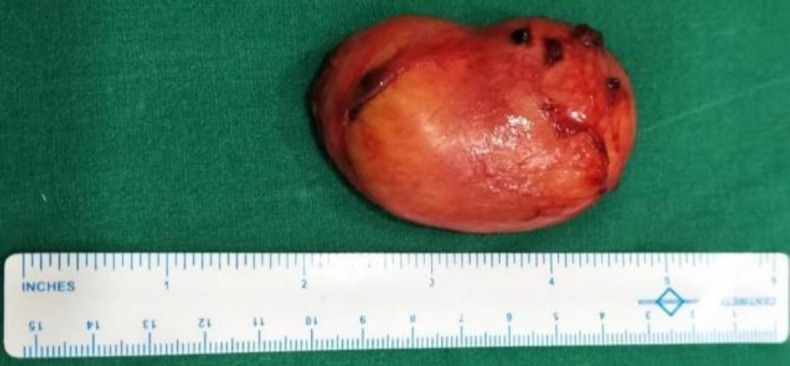
Sublingual schwannoma appearance after removal from the tongue

The diagnosis of schwannoma was confirmed with histological and immunohistochemical examinations. On physical examination, the excised mass exhibited a mucosa-covered yellow to white polypoidal nodular appearance (Fig.3). 

**Fig 4:**
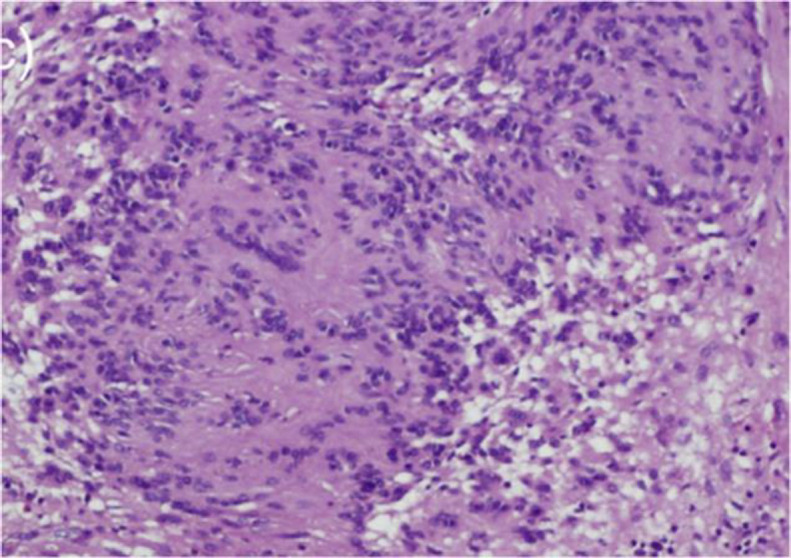
Schwannoma of tongue showing spindle cells arranged in palisading pattern and forming Verocay bodies (Antoni- A area). (H&E stain, 100X)

**Fig 5:**
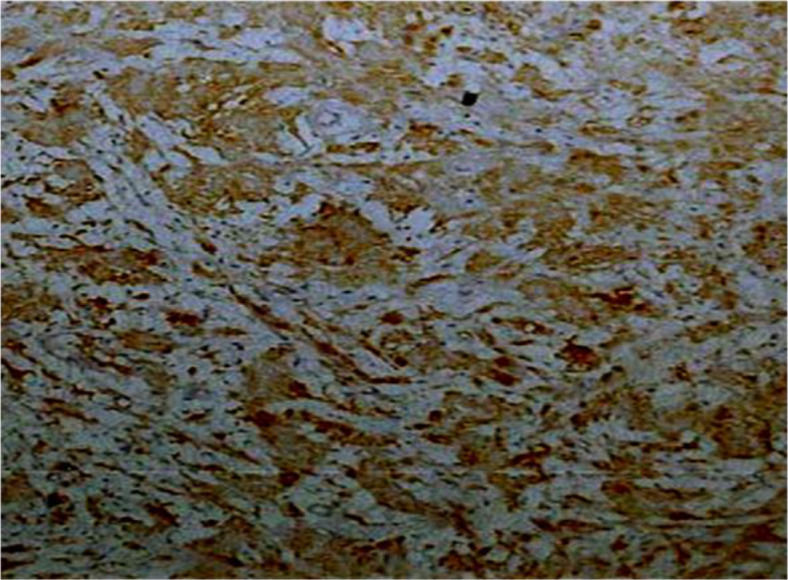
Schwannoma at the tongue showing strong S-100 reactivity (200X)

When the tumor was sliced apart, a smooth, pale white glistening neoplasm with hemorrhage streaks was discovered. This tumor was diagnosed histopathologically as a circumscribed, well-defined lesion with a thin mucosa lining. This neoplasm is made up of spindle-shaped Schwann cells that alternate between hypocellular and hypercellular regions. The hypercellular (Antoni A) areas had cellular interlacing eosinophilic patches (Fig.4), which are typical of Verocay bodies. The hypocellular (Antoni B) areas had a myxoid transformation. S100 positivity was found in spindle tumor cells by immunohistochemistry (Fig.5). 

The tumor cells were negative for cytokeratin and SMA, and Ki67 staining indicated a low proliferation index. As a result, it was identified as a benign schwannoma originating from the tongue's sublingual surface. The patient showed no signs of recurrence six months and a year after surgery. 

## Discussion

Schwannomas are benign tumors that originate from Schwann cells in peripheral, cranial, or autonomic nerves. In the oral cavity, these tumors are exceedingly uncommon. Schwannoma is one of these tumors (neurilemmoma or neurinomas), neurofibromas, and traumatic neuroma. Majority of the schwannomas at the head and neck region present as slowly enlarging, solitary, non-tender, and encapsulated mass. Schwannomas are benign tumors that arise from spinal roots, cervical nerves, sympathetic nerves, vagus, peroneal, and ulnar nerves and are encapsulated by the surrounding tissue. In neurofibromas, Schwann cells, neuritis, and fibroblasts are present inside a collagenous matrix (6). Schwannomas can develop on every cranial nerve save the first and second, with the 8^th^ cranial nerve being the most commonly affected. The roots of the spinal nerves, the flexor surfaces of the lower and upper limbs, and the tongue are among the other places where it may be found (7). Even though the majority of schwannomas are solitary, neurofibromatosis type 2 and schwannomas have been linked (8). Extracranially, the head and neck area accounts for around 25% of all schwannomas, while the intraoral region accounts for only 1%. (9). The tongue, palate, buccal mucosa, lips, and jaw are the most common sites for these intraoral tumors. Unless they grow to a significant size, these tumors are usually asymptomatic. Schwannomas are most common in people between the ages of 20 and 50, and they affect both men and women in similar numbers (10). The hypoglossal nerve is the most common source of tongue schwannomas. Typically, this neoplasm is asymptomatic, and it might go unnoticed for years before being diagnosed. Because of their enormous size and strain on the afflicted nerve, these tumors become symptomatic (11). It grows slowly, has a smooth surface, and is typically asymptomatic. This tumor is frequently a solitary one. However, it often presented with a painless mass in the tongue or at the sublingual area. When the mass is bigger, may present with pain, dysphagia, and dysphonia. The locations of the schwannoma of the tongue may be found at the anterior, posterior, anterolateral, ventral, dorsal, and tip of the tongue. In this case, the schwannoma of the tongue was very large with size 4.5x7 cm and located at the sublingual part i.e. ventral portion of the anterior part of the tongue. The sizes and locations of different cases of sublingual schwannoma published previously are given in Table.1. Although this tumor is generally solitary, it can be numerous or found in the context of von-neurofibromatosis, Recklinghausen's which virtually never progresses to cancer. A schwannoma can affect any region of the tongue. Robert et al. and Meyio et al. both found schwannoma on the tongue's ventral surface (12). Other studies have discovered schwannoma near the base and tip of the tongue. (13). 

The schwannoma in our instance is limited to the sublingual region of the tongue. Soft tissue tumors, malignancy, and salivary gland tumors are among the differential diagnosis for lingual schwannoma. Spindle cell lesions, neurofibromas, traumatic neuroma, squamous cell carcinoma, sarcoma, mucocele, fibroma, salivary gland neoplasms, smooth muscle tumors, fibroblastic neoplasms, lipoma, and ranula are among the differential diagnoses (15). 

**Table 1:** Sublingual schwannoma reported by different authors

**Author**	**Year**	**Patient age**	**Gender**	**Size(cm)**	**Location**	**Clinical presentations**	**Surgical approach**
Sethi et al.(21)	2008	28	Female	1	Anterolateral/ventral	Lump	Transoral
Gupta et al.(22)	2009	18	Female	1	Anterior/ventral	Lump	Transorsl
Cigdem et al.(23)	2010	13	Male	2	Anterior/ventral	Lump	Transoral
Moreno-Garcia et al.(24)	2014	13	Female	2	Anterior/ventral	Lump	Lip split/mandibulotomy
Bhola et al.(25)	2014	14	Female	1.5	Anteroateral/ventral	Lump	Transoral
Kavcic and Bozic.(26)	2016	20	Female	1.3	Anterorlateral/ventral/tip	Lump	Transoral
							

A systematic approach is required since radiology is an essential part of the assessment of tongue pathology. Because it provides for accurate localization of the tumor, greater visualization of the surrounding connections, and precise assessment of the tumor's size, MRI is the imaging of choice for lingual schwannomas. On T1W1 and T2W1, MRI indicates a well-circumscribed tumor with homogeneously isointense muscle and homogeneously hyperintense muscle. It also exhibits uniform amplification following contrast injection. On a contrast CT scan, schwannoma of the tongue exhibits heterogeneous enhancement (16). The tongue might also display cystic and fatty degeneration. The histological diagnosis of schwannoma is usually apparent because of the presence of alternating patterns of Antoni A and B areas, nuclear palisading, verocay bodies, and the whirling of cells. Histologically, meningioma, leiomyoma/ leiomyosarcoma, palisaded fibroblastoma, and pleomorphic hyalinizing angioectatic soft tissue tumors are comparable to neurilemmomas (11). 

Schwannomas have a high immunoreactivity for the protein S-100, which is used to diagnose them (17). 

Confirmation of the diagnosis can be done using histopathology and immunehisto- chemistry. The standard treatment for schwannoma, including tongue schwannoma, is the full surgical removal of the tumor (18). Based on the size and location of the tumor, surgical therapy for schwannoma in the oral cavity might follow either an intraoral or extraoral approach. In literature, the commonest approach for removal of the sublingual schwannoma was the transoral route (Table.1). In our case, the tumor is located at the sublingual area which is surgically excised via a transoral approach. In addition, if the schwannoma is particularly big, a preoperative examination of the airway is required. In the event of intraoral schwannoma surgery under general anesthesia, nasotracheal intubation is preferable for better tumor evaluation and excision. After full excision, no recurrence of the intraoral lingual schwannoma has ever been documented (3). Despite the rarity of malignant transformation, long-term monitoring is usually necessary (19,20). There are variations of locations and sizes of the intraoral sublingual schwannoma reported by different authors (21-26). Majority of these cases required transoral approach for complete excision of the sublingual schwannoma except one with lip split/ mandibulotomy (24).

## Conclusion

In regular clinical practice, schwannoma of the sublingual region is exceedingly rare. In benign sublingual lesions such as salivary gland tumors and ranula, it can be used as a differential diagnosis. After histological study and immunohistochemical investigation, the final diagnosis is verified. The best therapy for this tumor is total excision, which has a good prognosis. After full surgical excision, these tumors typically do not recur.
